# Basophils and Eosinophils in Nematode Infections

**DOI:** 10.3389/fimmu.2020.583824

**Published:** 2020-11-27

**Authors:** Kazushige Obata-Ninomiya, Phillip P. Domeier, Steven F. Ziegler

**Affiliations:** ^1^Immunology Program, Benaroya Research Institute, Seattle, WA, United States; ^2^Department of Immunology, University of Washington School of Medicine, Seattle, WA, United States

**Keywords:** helminth, nematode, allergy, basophil, eosinophil, Th2, type 2 immunity, Type 2 epithelial cytokines

## Abstract

Helminths remain one of the most prolific pathogens in the world. Following infection helminths interact with various epithelial cell surfaces, including skin, lung, and gut. Recent works have shown that epithelial cells produce a series of cytokines such as TSLP, IL-33, and IL-25 that lead to the induction of innate and acquired type 2 immune responses, which we named Type 2 epithelial cytokines. Although basophils and eosinophils are relatively rare granulocytes under normal conditions (0.5% and 5% in peripheral blood, respectively), both are found with increased frequency in type 2 immunity, including allergy and helminth infections. Recent reports showed that basophils and eosinophils not only express effector functions in type 2 immune reactions, but also manipulate the response toward helminths. Furthermore, basophils and eosinophils play non-redundant roles in distinct responses against various nematodes, providing the potential to intervene at different stages of nematode infection. These findings would be helpful to establish vaccination or therapeutic drugs against nematode infections.

## Introduction

Basophils and eosinophils, first described by Paul Ehlrich in 1879, are granulocytes (1, 2). Basophils and eosinophils are relatively rare when compared to other leukocytes. Basophils and eosinophils predominantly exist at most 0.5% and 5%, respectively, in peripheral blood under normal conditions, and have short half-lives when compared to lymphocytes. Intriguingly, basophils and eosinophils are evolutionally conserved in many animal species, suggesting their crucial and beneficial roles *in vivo*.

Basophils share some features with tissue-resident mast cells, which are abundant in peripheral tissues and long-lived cells. Basophils and mast cells are characterized by the expression of basophilic granules, surface expression of FcεRI, a high affinity IgE receptor, and release of chemical mediators (i.e., histamine) in response to cross-linking of surface IgE binding to FcεRI by antigens. Eosinophils have eosinophilic granules that contain eosinophil peroxidase (EPO), major basic protein, ribonulease cationic protein and eosinophil-derived neurotoxin, which are associated with allergic disorders and protection against parasites. In addition, Interleukin-5 receptor subunit-α on eosinophils define their unique biology in response to IL-5 produced by ILC2 and memory Th2 cells. Despite rarities of basophils and eosinophils in homeostatic conditions, basophils and eosinophils are found with increased their frequencies in peripheral tissues and play nonredundant roles in type 2 immune responses such as allergic inflammation and helminth infections. In the past 15 years, several works using deficient mice and specific-Cre mice for basophils or eosinophils characterized the indispensable roles of basophils and eosinophils in pathophisiology. In this review, we summarize the latest research on the pivotal and nonredundant roles of basophils and eosinophils in nematode infection. This review would be helpful to establish vaccination or therapeutic drugs against nematode infections.

## Development of Basophils and Eosinophils

Granulocytes develop from pluripotent CD34^+^ granulocyte progenitor (GP) cells in bone marrow through granulocyte/monocye progenitors (GMPs). GMPs derive the eosinophil lineage-committed progenitors (EoPs), and pre-basophil and mast cell progenitors (pre-BMPs) in bone marrow and the basophil/mast cell progenitors (BMCPs) in spleen. The pre-BMPs and BMCPs give rise to the mast cell progenitors (MCPs) and the basophil progenitors (BaPs) (3, 4) Controversially, Mukai et al. mentioned that BMCPs developed into mast cells, and not into basophils (5). The differentiation of basophils is regulated by Signal transducer and activator of transcription 5 (STAT5), the transcription factor distal promoter-derived Runt-related transcription factor 1 (Runx1), Interferon Regulatory factor 8 (IRF8), GATA binding protein 1 (GATA-1), GATA-2, and CCAAT/Enhancer Binding Protein α (C/EBPα) (5–10). STAT5 signaling is required for the differentiation of pre-BMPs into both basophils and mast cells through induction of GATA2, C/EBPα, and Microphthalmia-Associated Transcription Factor (MITF) that is important for differentiation of mast cells. Runx1-deficient mice exhibit a reduction of BaPs and basophils. Expression of IRF8 in GPs that assumingly develop from GMP to give rise to pre-BMP and BMCPs, is important for development of basophils upstream of GATA-2. The differentiation of eosinophils is regulated by GATA binding protein 1 (GATA-1), PU.1, and the CCAAT-enhancing binding protein (c/EBP) family of transcription factors. GATA-1 and PU.1 synergistically promote transcription of major basic protein (MBP). The absence of both MBP and EPO resulted in near complete loss of eosinophils *in vivo* (11).

GATA-1 reprograms immature myeloid cells to develop three different hematopoietic progenitor lineages: erythroid cells, megakaryocytes and granulocytes. GATA-1 is essential for maturation of erythroid and megakaryocyte precursors and positive autoregulation of GATA-1 expression is mediated by high affinity palindromic GATA-binding sites in the GATA-1 promoter (12, 13). Deletion of these GATA-binding sites in mice (called ΔdblGATA mice) results in a complete ablation of mature eosinophils (14). ΔdblGATA mice exhibit normal platelet development, and red blood cell production is only subtly impaired, but GATA-1 null mice have an embryonic lethal phenotype, with profound anemia and defective megakaryocyte development. As a result of these findings, ΔdblGATA mice were used as model of “eosinophil-deficient” mice, but later studies have defined additional roles for GATA-1 in the development of basophils and mast cells (15). GATA-1 expression is involved in the development and activity of megakaryocyte/erythrocyte progenitors, basophil/mast cell progenitors, basophil progenitors, mast cell progenitors and eosinophil progenitors but not granulocyte/monocyte progenitors (16–19). More recent studies have shown that ΔdblGATA mice exhibit additional defects in the generation of basophil precursor cells (BaP) and mature basophils (3, 20). Furthermore, basophils that do develop in ΔdblGATA mice have impaired IL-4 production and CD63 expression after cross-linking of antigen-specific IgE. Knockdown of GATA-1 in basophils *in vitro* resulted in defective basophil development, reduced degranulation and lower production of IL-4 in response to antigen stimulation. These suggested that defects in basophils of ΔdblGATA mice are due to decreased expression of GATA-1. In contrast to basophils, mast cell development in ΔdblGATA mice is not overtly impacted (21, 22). Similar to this, GATA-1-deletion does not affect development of mast cells *in vivo* and *in vitro* (23, 24). Collectively, ΔdblGATA mice showed developmental and functional impairments in basophils and eosinophils. In addition, the transcription factor GATA-1 controls both basophils and eosinophils.

## Basophils

### Basophilia in Parasite Infection

Although basophils make up a small proportion (<0.5%) of leukocytes in the blood, they accumulate in peripheral tissues during type 2 inflammation. Infiltration of basophils is observed in local lesions after helminth infection, and allergic skin diseases, implying that they may play important roles in supporting the inflammation (25, 26). Similar to allergic diseases, basophils accumulate in skin lesions of humans and mice after infestation with ectoparasites (27–29). However, unlike mice, blood basophilia rarely occurs in humans following nematode infections (30).

CD4^+^ T cell-derived IL-3 is critical for the survival and proliferation of basophils during a nematode infection (31). IL-3 activates basophils to produce IL-4 through IL-3Rα chain and FcR*γ* chain complex (32). Thymic stromal lymphopoietin (TSLP) induced by helminth infection, supports basophil proliferation and promotes induction of Th2 cytokine responses in *Trichinella* infection (33). During *Heligmosomoides polygyrus* (Hp) infection, IL-3, IgG1, and IgE selectively promote basophil expansion (34). IgE signaling promotes IL-3Rα chain expression on basophils (35). The factors that drive basophil expansion downstream of the IgE/FcεRI axis are still unknown. In mast cells, IgE induces survival *via* binding to FcεRI on mast cells by signaling through Bfl-1, a Bcl-2 family protein. However, the IgE/FcεRI/Bfl-1 axis apparently is not operative in human basophils (36, 37).

### Basophils and Type 2 Epithelial Cytokines

TSLP, IL-33, and IL-25 are predominantly produced from barrier epithelial cells to initiate type 2 immune responses, including eosinophilia. Thus, they are referred to as Type 2 epithelial cytokines.

Basophils express receptors for TSLP and IL-33 (38). TSLP activates basophils to produce IL-4, resulted in establishment of Th2 cell-dependent immunity (38). IL-33 activates basophils and mast cells to enhance the degranulation and production of cytokines such as IL-4, IL-6, and IL-13 (39). IL-33-mediated basophil activation has been discussed in atopic dermatitis (40). Single Nucleotide Polymorphisms (SNPs) in both *TSLP* and *CRLF2* coding TSLP receptor result in increased expression or signaling, and have been associated with Eosinophilic esophagitis (EoE) (41). In addition, IL-33 cytokine and receptor (*IL1RL1*, ST2) signaling is elevated in gastrointestinal allergic diseases, including food allergy and EoE (42). Of note, ST2 expression on basophils is necessary for basophil accumulation in the esophagus and the development of experimental EoE. Basophils are also required for TSLP-mediated EoE and IL-33-mediated food allergy in mice (43, 44).

The role of basophils in TSLP-dependent inflammation has been studied well, using topical vitamin D3 analog (MC903)-induced model of atopic dermatitis. Basophil-specific IL-4-deficient mice (IL-4 3’UTR mice) exhibit impaired ear swelling, reduced levels of antigen-specific serum IgE and diminished production of type 2 cytokines in lymph nodes after topical MC903 treatment (21). In addition, TSLP-stimulated basophils enhance ILC2 responses through production of IL-4, resulting in skin inflammation (45). However, basophil-specific TSLPR-deficiency by bone marrow chimerism does not confer protection from cutaneous inflammation or limit serum IgE titers after topical MC903 treatment (46). Furthermore, Basophil-specific TSLPR-deficiency in *Mcpt8^cre^Tslpr^fl/fl^* mice, did not impair the severity of the airway inflammation, generation of Th2 cells or levels of serum IgE when compared to control mice after intranasal challenges of antigen with MC903, suggesting that this type 2 inflammatory response was mediated by TSLPR on DC and CD4^+^ T cells, but not basophils and ILC2 cells (47).

### Basophils and Th2 Differentiation in Helminth Infection

Basophils promote Th2 cell differentiation through IL-4 production during *Trichinella spiraris* (Ts), *Heligmosomoides polygyrus* (Hp) and *Litomosomoides sigmodontis* Filaria infections (33, 48, 49). Giacomin et al. showed that deficiency of TSLPR, but not IL-3R, impaired basophilia in draining lymph nodes during Ts infection, which is associated with reduction of Th2 cells. Also, Th2 cell-mediated immune responses are important for expulsion of Hp parasites during re-infection (50).

Naïve CD4^+^ T cells require the interaction with peptide-loaded MHC class II (MHC-II) complexes on antigen presenting cells (APCs) to differentiate into Th2 cells, so Th2-differentiation could be primed by basophils. Pioneering work by Hida et al. showed that basophils produce IL-4 to support APCs, and promote Th2 cell differentiation (51). This finding was supported by follow-up studies from other research groups (52, 53). Later, three independent groups observed that basophils express MHC-II and secrete IL-4 to induce the differentiation of naïve CD4^+^ T cells to Th2 cells. Furthermore, depletion of basophils by anti-FcεRIα antibody (clone MAR-1) diminished Th2 cell differentiation *in vivo*. These findings suggest that basophils are professional APCs that express peptide-loaded MHC-II, induce Th2 differentiation in a cysteine protease papain-administration model, IgE and antigen-induced model and *Trichuris muris* (Tm) in primary infection (54–56). Yoshimoto et al. also showed that human basophils express MHC-II in that paper. However, the role of basophils as APCs is still under discussion. (1) Although all three papers used anti-FcεRIα antibody MAR-1 antibodies to deplete basophils *in vivo*, recent work revealed that MAR-1 binding is not limited to FcεRIα, but this antibody can also non-specifically bind to Fc*γ*RI and Fc*γ*RIV (57). Furthermore, treatment of MAR-1 depletes CD11c^+^ inflammatory DC *in vivo* (57–59). (2) When compared to DCs and B cells, basophils express low levels of surface MHC-II. Basophils also do not express the proteins that are required for MHC-II-restricted antigen processing or presentation, including cathepsin S, H-2M and the invariant chain Ii, and they exhibit a minimal capacity to process and present antigen when compared with DCs (52, 53, 58, 59). Miyake et al. demonstrated that basophils acquire peptide-MHC-II complexes from DCs *via* trogocytosis to prime Th2 cells in MC903 treatment–induced atopic dermatitis model (60). To resolve these remaining discrepancies, further studies will be needed to compare MHC-II functions on DCs and basophils with CD11c-Cre and basophil-specific Cre mice, respectively. Although some of basophil-specific Cre was based on a gene of mMCP-8, the technical caveat is that mMCP-8 expression is not restricted to basophils (61, 62).

Basophils are important for the expulsion of Tm from the cecum in primary infection. In the Tm model of helminth infection, Webb LM et al. showed that basophil localization to the caecum, but not the spleen, is regulated by Notch expression (63). Thus, Notch signaling in basophils is critical to induction of type 2 immune responses, including Th2 cell generation, RELM-β expression in cecum and resulting Tm clearance.

Recently, it has been reported that basophils can inhibit the type 2 immune responses *via* increase of expression of neuromedin B (NMB) receptors on ILC2 cells in primary infection of Nb, suggesting that basophils are not always inducer or enhancer of Type 2 immune responses (64).

### Basophils and M2-Type Macrophages in Helminth Infection

M2-type macrophages play key roles in regulating allergy and confer protective roles against helminths (50, 65–67). M2-type macrophages also protect against fatal lung damage in primary Nb infection (68).

**Figure 1:**
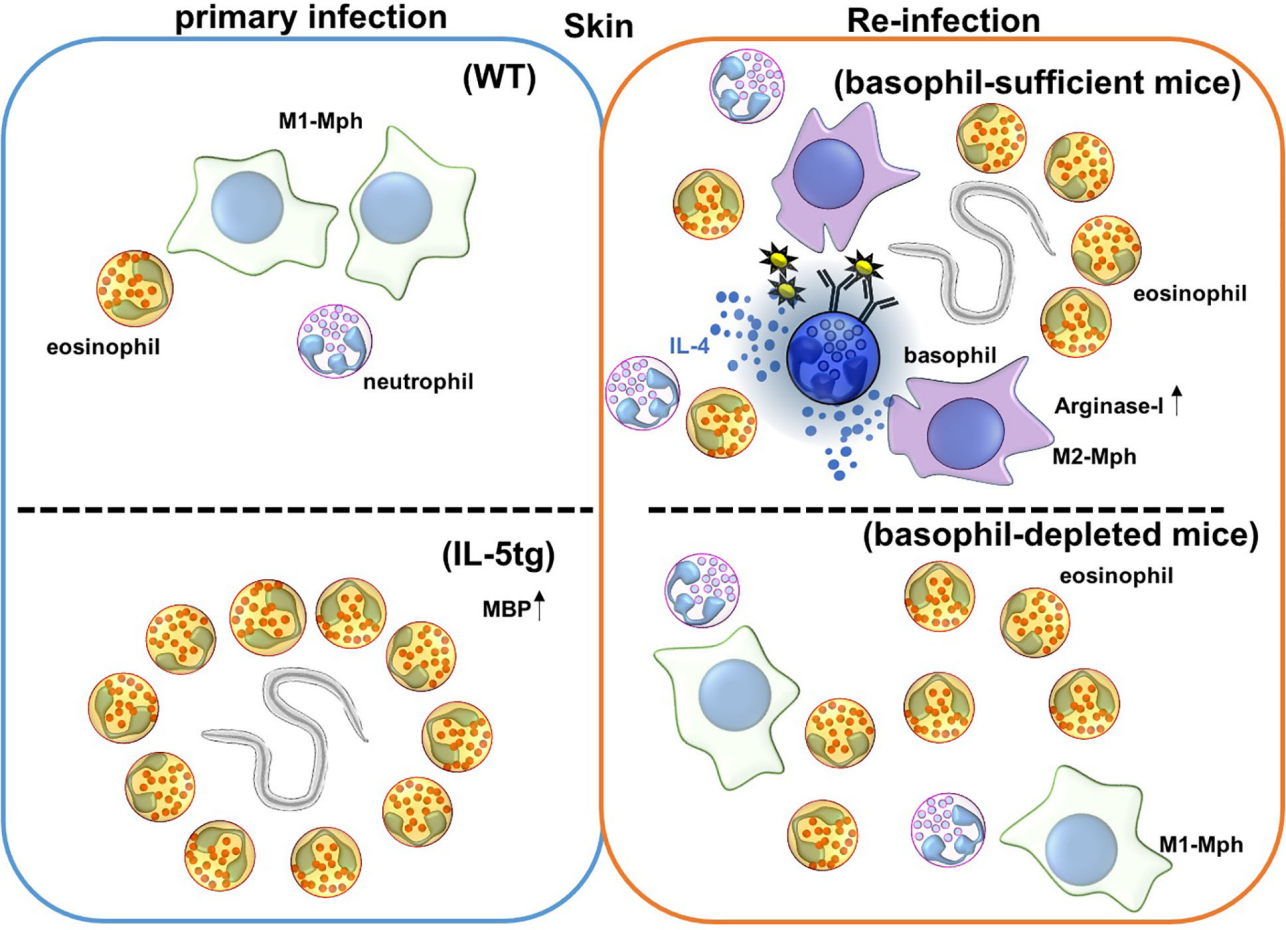
Immune responses against *Nippostrongylus brasiliensis* (Nb) infection in the skin. Nb leaves the skin and enters the blood and lymphatic vessels within 20 minutes of primary infection. In IL-5 transgenic mice, eosinophils are activated by overexpression of IL-5, and produce the anti-helminthic enzyme, major basic protein (MBP), causing Nb parasites to arrest in the skin lesion during primary infection. However, Nb antigens activate basophils during secondary infection, and correspondingly activate monocytes/macrophages to promote M2-type macrophage differentiation. Activated M2-type macrophages then secrete the anti-nematode enzyme, Arginase-I. Eosinophils are also accumulated in the skin, but the role of these accumulated eosinophils is not yet known. Depletion of basophils allows for Nb parasites to leave the skin, but eosinophils still infiltrate the tissue during secondary infection.

Basophil depletion by Thy1.2 or CD200R3 antibody correlates with increased worm burden after re-infection with *Nippostrongylus brasiliensis* (Nb) (69–71) (**Figure 1** and **Table 1**), and this protection is mediated by basophil-derived type 2 cytokines (72). During Nb infection, cross-linking of Nb antigen-specific IgE promotes basophil activation and IL-4 production. Basophil-derived IL-4 promotes M2-type macrophage differentiation, and the production of anti-parasitic enzyme Arginase-1 to protect against secondary Nb infection in the skin (73). Similar to this, Depletion of macrophages expressing *Relma* that is a marker of M2-type macrophages increased worm burden in the lungs and gut (68). Conversely, basophil-deficiency did not affect the protection against a secondary subcutaneous inoculation of *Strongyloides venezuelensis* (Sv) or *Strongyloides ratti* (Sr), which originally penetrate skin to infect the hosts by a mechanism that is similar to Nb (74–76) (**Table 2**).

**Table 1:** The experimental models of nematode infections.

Helminth	Infection stage	Natural Route of infection	Route of Experimental inoculation	Characteristics of Infection	Model for infection in human (infection route)
*Nippostrongylus brasiliensis*	L3 larvae	Skin penetration	i.d., s.c.	Naturally penetrate the skin and migrate to the lungs. Parasites then migrate to the gut to lay eggs. Short-lived infection	*Ascaris lumbricoides* (Oral ingestion)
					Hookworms;
*Strongyloides venezuelensis*	L3 larvae	Skin penetration	s.c.		*Necator americanus* (Oral ingestion),
*Strongyloides ratti*					*Ancylostoma duodenale* (Oral ingestion or skin penetration)
					Strongyloides stercolaris (Skin penetration)
*Heligmosomoides polygyrus*	L3 larvae	Oral ingestion	p.o.	Chronic infection from ingestion of larvae	
*Trichinella spiralis*	L1 larvae	Oral ingestion	p.o.	Food-borne (infective juvenile), zoonotic parasite	*Trichinella spiralis* (Oral ingestion)
*Trichuris muris*	Eggs	Oral ingestion	p.o.	Ingestion of infectious eggs that hatch in the cecum and colon	*Trichuris trichiura* (Oral ingestion)
*Litomosoides sigmodontis*	L3 larvae	mite	s.c., mite	Chronic infection	Human filarial diseases;
*Brugia pahangi*	L3 larvae	mosquito	s.c.	Adult worms inhabit the pleural cavity	*Brugia malayi*, *Brugia timori*,
					*Wuchereria bancrofti*
					*Onchocerca volvulus*,
					*Loa loa*

**Table 2:** The role of basophils and eosinophils in nematode infections.

Helminth	The role of Basophils	The role of eosinophils	Reference
Nippostrongylus brasiliensis	- Basophils protect from re-infection in the skin.	- CXCR6^+^ST2^+^ mTh2 cells facilitate eosinophilia in the lungs to reduce the fecundity in the lungs in re-infection.	(73)
			(77)
*Strongyloides venezuelensis*	- Basophil-depletion in Mcpt8DTR mice revealed small contribution of basophils in primary infection and minor or no roles in secondary infection.	- The duration of Sv was increased in ΔdblGATA mice in primary infection (unpublished data)	(75)
			(74)
*Strongyloides ratti*	- The number of intestinal nematodes and fecal eggs is elevated in Mcpt8-Cre mice.	- IL-5 deficiency increased the number of intestinal worms and fecal eggs.	(76)
			(78)
Heligmosomoides polygyrus	- Mcpt8-Cre mice have a high number of eggs in feces during re-infection.	- The fecundity of Hp was increased in ΔdblGATA and PHIL mice during re-infection.	(48)
			(79)
*Trichinella spiralis*	- Th2 immune response is reduced in Bas-TRECK mice.	- Eosinophils increased the survival of muscle larvae	(33)
			(80)
*Trichuris muris*	- Basophil depletion *via* MAR-1 treatment increases the number of Th2 cells and impairs Tm expulsion.	- Eosinophil depletion by anti-IL-5 Ab treatment does not change worm expulsion.	(54)
			(81)

M2-type macrophages also provide protection against secondary infection of *Heligmosomoides polygyrus* (Hp), but M2-type macrophage differentiation during Hp infection is induced by IL-4 from CD4^+^ T cells in small intestine (50). However, the expression of FcR, IL-4 and IL-13 on basophils is required for Th2 cell priming, downstream M2-type macrophage differentiation and Hp worm clearance (48). Taken together, after surface-bound IgE is cross-linked by helminth-derived antigens, basophils produce IL-4 and IL-13 to induce M2-type macrophage differentiation, resulting in expulsion of Nb and Hp from the skin and small intestine.

Non-basophils can also produce type 2 cytokines to induce M2-type macrophages in protection against Nb re-infection in the lungs (67, 82). As described by Chen et al. neutrophils in Nb-infected mice upregulated IL-13 transcripts in secondary infection, suggesting that neutrophils could promote M2-type macrophage activation in the lungs to clear Nb parasites (67). Conversely, another study showed that ILC2 and Th2 cells, but not neutrophils, could potentially induce M2-type macrophage activation to kill Nb at the lung during secondary infection (82).

### Basophil-Derived Proteases

Basophil-derived proteases, including serine protease mouse mast cell protease-8 (mMCP-8), and tryptase mMCP-11, play an important role in promoting skin inflammation (83, 84). The mMCP-11 increases vascular permeability, allowing for increased migration of basophils, eosiniophils, macrophages and neutrophils. Intriguingly, mMCP-11-deficiency ameliorates IgE-mediated chronic allergic inflammation in the skin. Furthermore, intradermal administration of mMCP-8, induces the production of Cxcl1, Ccl2, and Ccl24, which recruit neutrophils, monocytes, and eosinophils into the lesion. Similar to atopic dermatitis, Nb re-infection is characterized by increased numbers of neutrophils, macrophages, eosinophils and basophils in the skin lesion and high titers of IgE in the serum. However, the role of these proteases in anti-helminth immunity is not yet known.

Basophils are involved in resistance against both *Strongyloides venezuelensis* (Sv) and *Strongyloides ratti* (Sr) in primary infection (75, 76). The contributions of basophils in induction and expansion of Th2 cells are negligible, although those parasites are expelled by type 2 immune responses from small intestine. It might be possible that basophil-specific molecules such as mMCP-8 and mMCP-11 are associated with the protection against these nematodes.

## Eosinophil

Eosinophils make up about 5% of leukocytes in peripheral blood, and have a short half-life in circulation. However, the number of circulating eosinophils are increased in patients with allergic diseases and helminth infections. Eosinophil granules contain major basic protein, eosinophil cationic protein, eosinophilic peroxidase and eosinophil-derived neurotoxin.

Approximately, 7–10% of the total protein content of human eosinophils consists of galectin-10, while mice do not contain a functional galectin-10 gene (85, 86). Upon Eosinophil activation, secreted galectin-10 protein forms aggregates, called Charcot-Leyden crystals, at sites of inflammation. Charcot-Leyden crystals were first described as extracellular bipyramidal crystals in the airways of patients with asthma in 1853 by Charcot, and this observation was confirmed by Leyden in 1872. However, the link between Charcot-Leyden crystals and eosinophilic airway disease and/or mucus production was largely forgotten for over 100 years. Recent work from Persson et al. showed that intratracheal administration of galectin-10 promoted the infiltration of neutrophils and monocytes, and Th2 cell priming (87).

GM-CSF, IL-3, and IL-5 accelerate the growth, maturation, survival, and activation of eosinophils. GM-CSF-deficient mice exhibit impaired recruitment of eosinophils to airways in a model of allergic airway. IL-5 deficiency is correlated with a 2- to 3-fold reduction in B-1 cells and eosinophils as compared control mice. However, the eosinophils that did develop in IL-5–deficient mice were morphologically similar to eosinophils in control mice, but IL-5–deficient mice failed to develop blood and tissue eosinophilia in response to helminth infection (88). IL-5 transgenic (IL-5tg) mice overexpressing IL-5 in homeostatic condition have elevated numbers of circulating eosinophils, neutrophils, lymphocytes and monocytes (89).

### Eosinophils and Type 2 Epithelial Cytokines

Type 2 epithelial cytokines activate ILC2 and Th2 cells to produce IL-5 and IL-13, leading to eosinophil infiltration in allergic inflammation and helminth infection (77, 90, 91).

It has been reported that eosinophils express own receptors for Type 2 epithelial cytokines. Human eosinophils express functional TSLP receptor components: TSLPR and IL-7Rα. TSLP up-regulates the expression of adhesion molecule CD18 and intercellular adhesion molecule-1, while down-regulating L-selectin, resulting in increased migration by eosinophils to promote tissue eosinophilia (92). TSLP also induces eosinophil degranulation and the release of eosinophil extracellular traps to capture extracellular bacteria (93). Although TSLP supports the survival of various leukocytes including T cells and non-hematopoietic cells, the role of TSLP in maintaining eosinophil survival is controversial (94–96). Two studies examined the role of TSLP in survival of eosinophils; while one reported to enhance survival of eosinophils, the other did not report a notable change in eosinophil survival (97, 98). These results suggest that eosinophils are involved in pathogen defense when TSLP production is triggered by environmental factors. Furthermore, tuft cells located in mucosal epithelial layer predominantly produce IL-25 to activate ILC2 cells (99). Tuft cells monitor the microbial metabolite succinate to initiate type 2 inflammation including tuft cell and goblet cell hyperplasia, and eosinophilia (100).

### Eosinophil in the Skin in Helminth Infections

**Figure 2:**
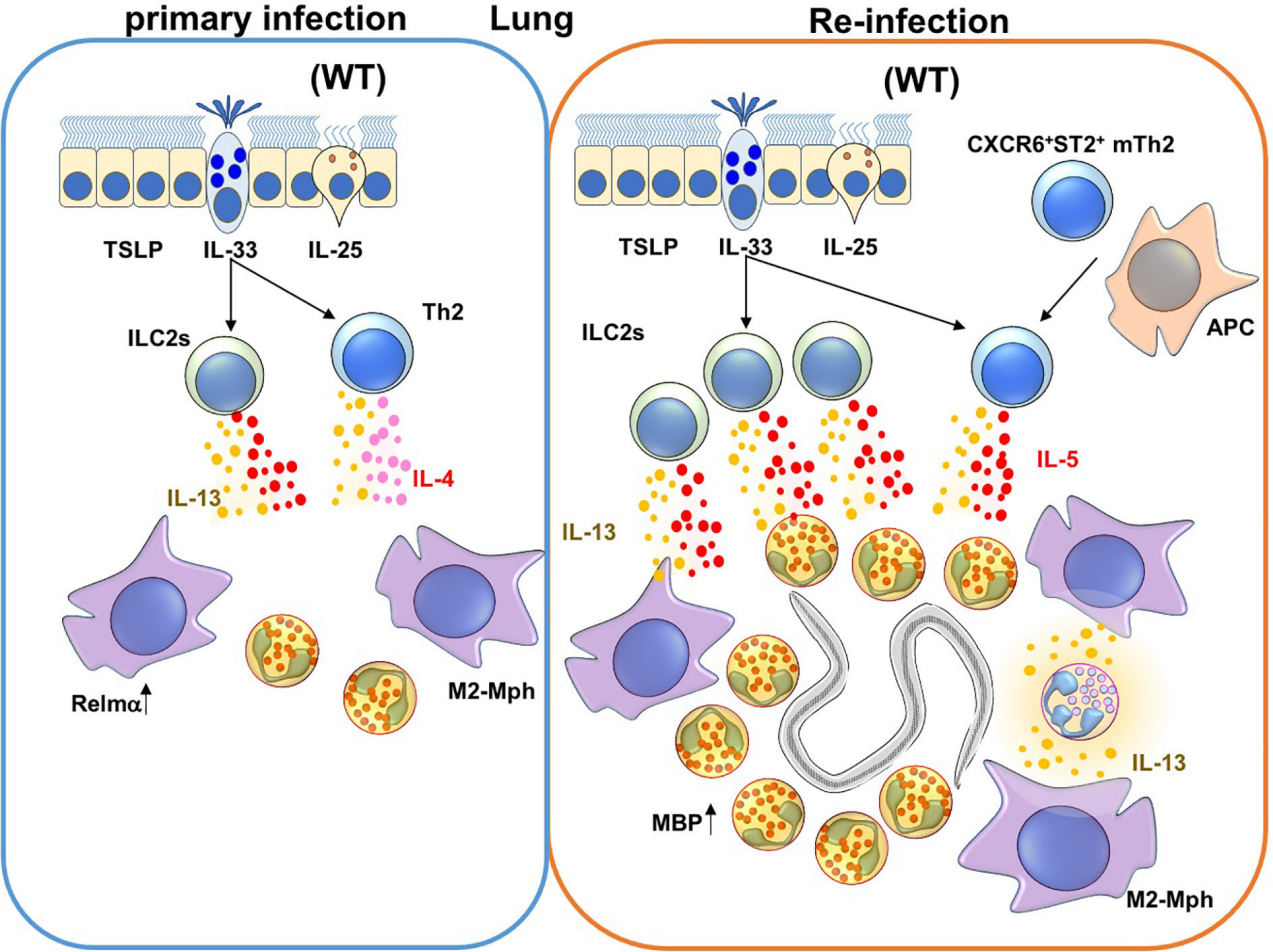
Immune responses against *Nippostrongylus brasiliensis* (Nb) infection in the lungs. During a primary Nb infection, Nb leaves the lungs before the peak of infiltration of eosinophils and M2-type macrophages to the site of lung infection (68). On the other hand, during re-infection of Nb, activated Innate Lymphoid Cell 2 cells (ILC2s) and CXCR6^+^ST2^+^ memory Th2 cells produce IL-5 and IL-13. IL-5 and IL-13 promote lung infiltration, activation and major basic protein (MBP) production by eosinophils. In addition, M2-type macrophages are also induced by type 2 cytokines that could be produced by ILC2s, Th2 cells and/or neutrophils in re-infection.

Eosinophilia in the skin occurs during re-infection with *Nippostrongylus brasiliensis* (Nb), while ΔdblGATA mice lacking eosinophils are susceptible to re-infection of Nb. Thus, eosinophils are believed to play an important role in providing protection during Nb re-infection (101, 102). As we mentioned above, since ΔdblGATA mice display numerical and functional aberrancy in basophils, adoptive transfer of wild-type basophils into ΔdblGATA mice confers the protective immunity against Nb in the skin in re-infection (20). Antibody-mediated depletion of eosinophils, with a combination of anti-IL-5 and anti-Siglec-F antibodies does not change the Nb parasite burden in the lungs, suggesting that basophils, rather than eosinophils, are primarily important for providing protection from Nb in skin in re-infection (77) (**Figure 2**). The role of eosinophils in the skin during Nb re-infection is not yet clear, but eosinophils have known roles in tissue repair and help the helminth infection. Eosinophils promote skin tissue repair by producing TGF-β during the resolving phase of inflammation (103). It has been also known that eosinophils promote *Trichinella spiralis* (Ts) infection; eosinophils help survival of Ts larva in the muscles of hosts (104). Eosinophils increase the fecundity of *Heligmosomoides polygyrus* (Hp), and reduce IL-4 response by follicular helper T cells and IgG1 class-switching in peyer’s patches in re-infection (79).

### Eosinophils in the Lungs in Helminth Infection

Eosinophils are recruited into Nb-infected lungs during primary and secondary infection by ILC2 and CXCR6^+^ST2^+^ memory Th2 cells (77, 82, 90, 105) (**Figure 1** and **Table 1**). ILC2 and CXCR6^+^ST2^+^ memory Th2 cells express IL-33 receptors and produce high concentrations of IL-5 and IL-13 during allergic responses and parasitic infections (91, 106, 107). Eosinophil-deficiency alone does not change the duration of primary Nb infection, but eosinophils are required to stall parasite maturation in the lung during re-infection with Nb (77, 108). Adoptive transfer of CXCR6^+^ST2^+^ memory Th2 cells from Nb-sensitized mice conferred resistance to Nb in the lungs of recipient mice. IL-5 is also required to induce major basic protein (MBP) secretion by eosinophils (77). Adoptive transfer of eosinophils, but not MBP-depleted eosinophils, into the lungs inhibited Nb maturation. MBP expression in eosinophils is also required for eosinophils to kill *Strongyloides stercoralis* (Ss) parasites in implanted cell-impermeable diffusion chambers (109). Collectively, these findings suggest that eosinophils protect from Nb re-infection in the lungs but not skin, and that MBP produced by eosinophils is required for protection against Nb and Ss.

IL-5 transgenic (IL-5tg) mice overexpressing IL-5 in homeostatic condition have elevated numbers of circulating eosinophils, neutrophils, lymphocytes and monocytes (89). Since IL-5tg mice exhibit a pronounced elevation of eosinophils, they are classically used to model chronic eosinophilia in mice. IL-5tg are strongly resistant to several helminth infections, including Nb and Ss (109, 110). Adoptive transfer of eosinophils from IL-5tg mice conferred the protection against Nb. These imply that high levels of IL-5 confer the capacity to protect from Nb infection to eosinophils (111). In the same context, Yasuda et al. demonstrated that Sv infection prior to Nb infection caused mice to acquire a highly responsive “trained” phenotype. This trained phenotype was associated with a reduction in the number of Nb larvae in the lungs as a result of an enhanced accumulation of ILC2 cells that produced IL-5 and IL-13 to promote pulmonary eosinophilia (107).

## Basophil, Eosinophils, and Antibody Production During Helminth Infection

B cells are required for protection from various infections of nematodes including *Strongyloides venezuelensis*, *Nippostrongylus brasiliensis* (Nb), *Trichuris muris* and *Heligmosomoides polygyrus* (48, 73, 74, 112), and both basophils and eosinophils are crucial for the production and maintenance of parasite-specific antibodies during *Trichinella spiraris* infection (49, 80). Early evidence suggests that basophils, CD4^+^ T cells and B cells provide interconnecting roles in the response to parasitic infection, but the mechanisms that coordinate these cells remain poorly understood.

Activated basophils express CD40 ligand and secrete IL-4 and IL-13, which are required for IgE class-switching and production during parasitic infection (113–116). However, this basophils activation is predominantly mediated by FcεRI cross-linking (117), suggesting that the capacity for basophils to activate B cell class-switch recombination occurs after the initial priming of parasite-specific B cells. Alternatively, after co-culture with basophils, CD4^+^ T cells exhibit an augmented capacity to induce IgE class-switching in B cells (118), so basophils could also prime IgE responses through a CD4^+^ T cell intermediate. After class-switching, basophils support the survival of plasma cells and memory B cells through the production of IL-4 and IL-6 in the spleen and bone marrow (118, 119), and eosinophils support the survival of plasma cells in the bone marrow through the secretion of IL-6 and APRIL (120).

IgE production relies on IL-4 production by follicular helper cells (Tfh) (121, 122). Tfh cells are divided into subsets by gene expression profiles and functional roles that mirror T-helper cell subsets in humans and mice; Tfh1, Tfh2, Tfh17, Tfh13, and follicular regulatory T (Tfr) cells (123–125). It has been reported that the generation of IL-4-expressing Tfh2 cells is facilitated by basophils in response to cross-linking of IgD on the basophil surface (126). More recently, Gowthaman et al. showed that a rare population of IL-13 producing Tfh (Tfh13) cells is required for the production of high affinity, anaphylactic IgE against allergens, whereas infection of Nb with OVA did not generate Tfh13 cells (125). However, Tfh13-induced IgE is regulated by Tfr cells in the germinal center, suggesting that Tfr cells could be limiting Tfh13 cells activity during Nb infection (127). The absence of Tfh13 in helminth infection could explain why high affinity IgE antibodies are not detected during helminth infection.

Collectively, basophils and eosinophils have the potential to contribute to the generation of IgE specific for helminth and helmith-derived antigens, and in turn, these antibodies coat FcεRI on basophils to arm them for rapid activation during re-infection of the helminth.

## Vaccination Against Helminth

*Litomosoides sigmodontis* (Ls) is a filarial nematode parasite that is used as a model of filarial diseases. The parasites are inoculated or transmitted through the skin barrier by mites, and they inhabit the pleural cavity after developing into adult worms. When irradiated parasites are injected into mice as a method of vaccination, protective immunity against Ls larvae is induced in a basophil-dependent manner, but basophils are dispensable as effector cells against live Ls (128). Vaccination of mice with *Heligmosomoides polygylus* (Hp)-excretory secretory products confers the resistance against Hp larvae, but protective immunity depends on neutrophils, but not eosinophils, basophils or mast cells. However, basophils (but not eosinophils) do contribute to the worm expulsion during secondary re-infection with Hp (48, 79, 129). Killing trapped parasites in the small intestine is partially dependent on eosinophils.

The adjuvant effects of TNF-α have been well documented. Mast cells produced TNF-α to orchestrate the recruitment of T cells and dendritic cells into draining lymph nodes in *Escherichia coli* or *Klebsiella pneumoniae* infections (130). Other studies showed that TNF-α and synthetic granules mimicking granules of mast cells can be used for vaccination (131). Recently, Piliponsky AM et al. published that basophil-derived TNF-α enhanced survival in a sepsis in mice (132). Together with this, it could be possible to use basophils as one of the primary targets for vaccination as the adjuvant function.

## Conclusion

Here, we summarized the roles of basophils and eosinophils in nematode infections. We also showed that granulocytes are stringently controlled by type 2 epithelial cytokines, and control type 2 immune responses by promoting Th2 cell differentiation and antibody production. Recent findings demonstrate that basophils and eosinophils are key players in protective immune responses against helminths. Although basophils and eosinophils are not primarily associated with directly killing nematodes during primary infection, these cells hinder parasite burden during reinfection by enacting the rapid deployment of type 2 immune responses. In response to nematode parasite re-infection, basophils are armed with IgE specific for nematodes or their products and accumulate in the peripheral tissues. After antigen stimulation, basophils secrete IL-4 to induce M2-type macrophages, and proteases to rapidly recruit monocytes, neutrophils, and eosinophils to the infection site. In response to IL-5, eosinophils are activated to a “trained” phenotype and produce major basic protein (MBP) to kill nematodes. On the other hand, eosinophils can also support nematode survival and tissue repair during the resolving phase. Further studies are necessary to fully characterize how basophils and eosinophils coordinate their cell-specific responses to expel nematodes. However, therapeutic targeting of basophils and eosinophils or their products could be crucial for developing novel therapeutic interventions against nematode infections.

## Author Contributions

KO-N, PPD and SFZ wrote the manuscript. All authors contributed to the article and approved the submitted version.

## Funding

Support for this work was provided by NIH grants AI068731, AI125378, AI124220, and HL098067 to SFZ.

## Conflict of Interest

The authors declare that the research was conducted in the absence of any commercial or financial relationships that could be construed as a potential conflict of interest.
